# Dry-grind processing using amylase corn and superior yeast to reduce the exogenous enzyme requirements in bioethanol production

**DOI:** 10.1186/s13068-016-0648-1

**Published:** 2016-10-24

**Authors:** Deepak Kumar, Vijay Singh

**Affiliations:** Department of Agricultural and Biological Engineering, University of Illinois at Urbana-Champaign, Urbana, IL 61801 USA

**Keywords:** Amylase corn, Bioethanol, Dry-grind, High-solid fermentation, In situ ethanol removal

## Abstract

**Background:**

Conventional corn dry-grind ethanol production process requires exogenous alpha and glucoamylases enzymes to breakdown starch into glucose, which is fermented to ethanol by yeast. This study evaluates the potential use of new genetically engineered corn and yeast, which can eliminate or minimize the use of these external enzymes, improve the economics and process efficiencies, and simplify the process. An approach of in situ ethanol removal during fermentation was also investigated for its potential to improve the efficiency of high-solid fermentation, which can significantly reduce the downstream ethanol and co-product recovery cost.

**Results:**

The fermentation of amylase corn (producing endogenous α-amylase) using conventional yeast and no addition of exogenous α-amylase resulted in ethanol concentration of 4.1 % higher compared to control treatment (conventional corn using exogenous α-amylase). Conventional corn processed with exogenous α-amylase and superior yeast (producing glucoamylase or GA) with no exogenous glucoamylase addition resulted in ethanol concentration similar to control treatment (conventional yeast with exogenous glucoamylase addition). Combination of amylase corn and superior yeast required only 25 % of recommended glucoamylase dose to complete fermentation and achieve ethanol concentration and yield similar to control treatment (conventional corn with exogenous α-amylase, conventional yeast with exogenous glucoamylase). Use of superior yeast with 50 % GA addition resulted in similar increases in yield for conventional or amylase corn of approximately 7 % compared to that of control treatment. Combination of amylase corn, superior yeast, and in situ ethanol removal resulted in a process that allowed complete fermentation of 40 % slurry solids with only 50 % of exogenous GA enzyme requirements and 64.6 % higher ethanol yield compared to that of conventional process.

**Conclusions:**

Use of amylase corn and superior yeast in the dry-grind processing industry can reduce the total external enzyme usage by more than 80 %, and combining their use with in situ removal of ethanol during fermentation allows efficient high-solid fermentation.

## Background

Due to increasing population and industrialization, global energy demand has increased steadily over the last few decades, and currently, about 80 % of this energy is derived from non-renewable fossil fuel supplies [1]. Transportation sector is one of the major consumers of the fossil fuels in the United States [2]. The concerns of depleting fossil fuel and the negative environmental impacts from their use necessitate the need to identify and develop renewable and sustainable energy sources. Bioethanol is considered as the most promising renewable transportation fuel, which can be produced in significant quantities from fermentation of sugars obtained from starch, sugary or cellulosic materials. United States is the biggest bioethanol producer in world with about 14.3 billion gallon (54.1 billion liters; 58 % of world production) production in year 2014 [3]. Most of the ethanol in the United States is produced from corn using dry-grind or wet milling process. Dry-grind is the most common used method for corn ethanol production [4]. In year 2014, about 5.4 billion bushels (25.4 kg in one bushel) of corn (37.8 % of total production) was processed in dry-grind industry [5].

**Fig. 1:**
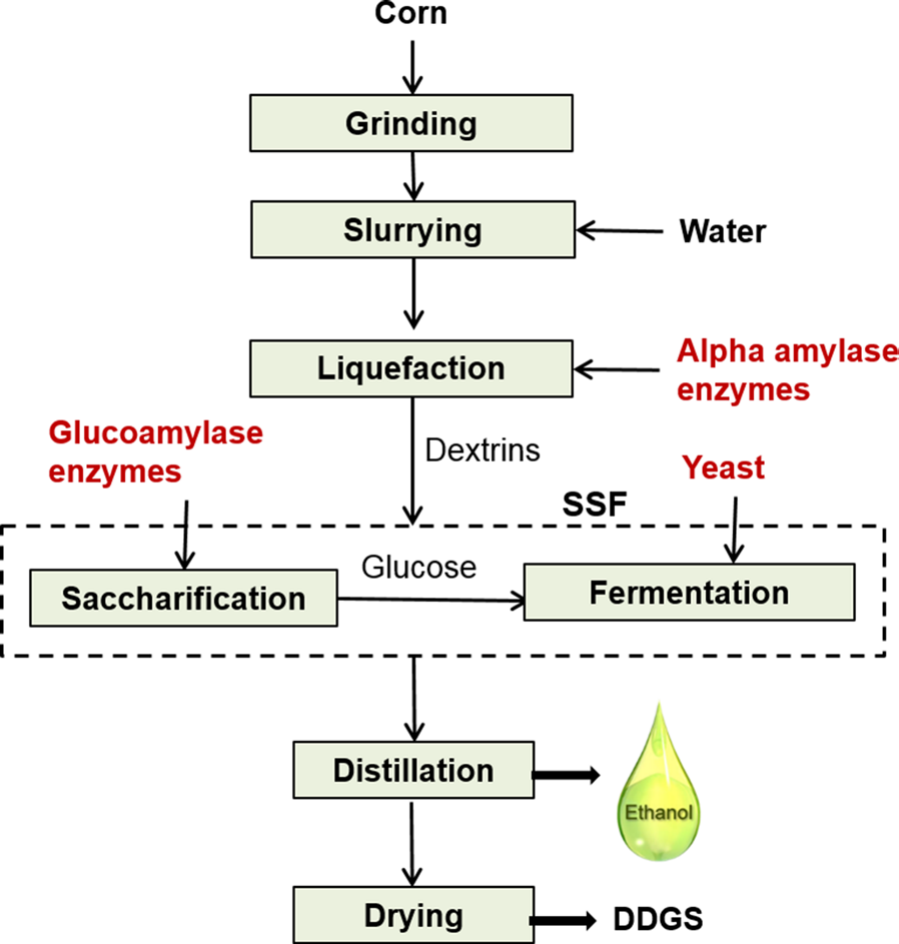
Schematic of laboratory scale dry-grind corn process for ethanol production. Figure illustrates the steps followed during lab scale dry-grind processing for ethanol production from corn

Figure 1 illustrates the major steps used during laboratory scale conventional dry-grind process. The ground corn and water slurry is liquefied using α-amylase enzymes at high temperatures to convert starch into dextrins. The dextrins are further converted to glucose using glucoamylase (GA) enzymes during saccharification process, which is fermented to ethanol by yeast. Currently, these alpha and glucoamylases enzymes are added externally in liquid form during the liquefaction and saccharification process respectively. Saccharification and fermentation are performed in single step in the same reactor by process known as simultaneous saccharification and fermentation (SSF). Ethanol is recovered from the fermentation broth using distillation process. Remaining non-carbohydrate fractions in corn (germ, fiber, and protein) are recovered as a co-product called distillers dried grains with soluble (DDGS) at the end of the process.

Over the last few decades, several advances have been made to improve the ethanol yields and profitability of the dry-grind process, including modifications in the production process [6], recovery of high-value co-products [5, 7], use of advanced enzymes [8, 9], and use of high-yield corn varieties [10].

A new corn developed by transgenic technology, known as amylase corn, produces an endogenous α-amylase in endosperm that is activated at high temperature and moisture [10, 11]. Due to high expression levels of enzymes, only a small amount of the corn is required to be mixed with the conventional dent corn. Use of the amylase corn mix during the dry-grind process can eliminate the need of external addition of exogenous α-amylase. Similarly, a new engineered yeast, referred as “superior yeast” in this manuscript, is an advanced strain of *Saccharomyces cerevisiae* which expresses endogenous glucoamylases and provides novel metabolic pathways for high ethanol yields by reducing glycerol production. Use of this yeast can eliminate or alleviate the addition of expensive glucoamylase enzymes during SSF process, potentially improving the process efficiency, and reducing the overall ethanol production cost.

Increasing the solid loadings during dry-grind process can be another approach to reduce the overall cost of ethanol production process. Using high-solid slurries in dry-grind process can decrease the overall energy use and process cost by reducing load on downstream processing of ethanol and co-product recovery and lowering the volumes of the processing equipment. However, the solid loadings during the ethanol process are restricted to 30–32 % w/w due to high viscosities, and yeast stress by high glucose and ethanol concentrations [12–14]. High-solid loadings can lead to higher final ethanol concentrations; however, low ethanol yields (liters/metric ton or gallons/bushel) are observed because of strong ethanol inhibition [15]. Simultaneous stripping off ethanol under vacuum during SSF process is one of the potential approaches to reduce the ethanol inhibition and achieve high-solid loadings [16]. With application of vacuum, ethanol can be evaporated at the normal fermentation temperature without affecting the yeast activity. Some studies on ethanol and butanol production have concluded that fermentation efficiencies can be improved significantly by applying only few cycles of vacuum [12, 13, 17].

Objectives of this work were to investigate the strategies to reduce external exogenous enzyme requirements during dry-grind process and improve ethanol yields at high-solid loadings. The fermentation characteristics of dent corn and amylase mix corn were evaluated using a superior yeast at various loadings of glucoamylase enzyme (0, 25, and 50 %), and the performance was compared with conventional yeast and glucoamylase used in the dry-grind process. The fermentation behavior of amylase mix corn using superior yeast was investigated using vacuum flashing process to achieve high ethanol yields by reducing ethanol inhibition at high-solid loadings.

## Methods

### Materials

Conventional yellow dent corn was generously donated by a commercial seed company (DuPont Pioneer). The amylase corn was obtained from another commercial seed company (Syngenta Biotechnology, Inc., Research Triangle Park, NC). Corn samples were hand-cleaned and sieved using a 12/64″ (4.8 mm) sieve to remove broken corn and foreign materials. The cleaned corn was stored in refrigerator at 4 °C till analysis. The moisture content in corn was determined by drying the samples in hot air oven at 135 °C for 2 h (AACC International Approved Method 44-19.01) [18]. Starch content in the ground corn flour was determined using enzymatic assay (AACC International Approved Method 76-13.01) using the Total Starch Kit (Megazyme, Bray, Co. Wicklow, Ireland) [18].

The α-amylase and glucoamylase employed in this study were commonly used commercial enzymes. The α-amylase enzyme has an activity of 6400 µmol maltose/min mL. The glucoamylase enzyme activity has been reported 775 AGU/mL. Conventional active dry yeast (ethanol red) was obtained from the Fermentis-Lesaffre Yeast Corporation (Milwaukee, Wisconsin). The superior yeast was provided by the Lallemand Biofuels and Distilled Spirits (Milwaukee, WI).

### Dry-grind process

The cleaned samples were ground in a laboratory scale hammer mill (model MHM4, Glen Mills, Clifton, NJ) at 500 rpm and using a 0.5-mm screen. Conventional dent corn and amylase corn were ground separately and later mixed to form a 15 % (by dry weight) amylase corn mixture, referred as “amylase mix corn” in this manuscript. All dry-grind experiments were performed at 250 mL scale in 500 mL stainless steel reactors in triplicate. Ground corn was mixed with deionized (D.I.) water to make slurry having 30 % solids on dry basis. For the liquefaction of control samples (100 % dent corn), the pH of the slurry was adjusted to 5.1 using 10 N sulfuric acid and 25.7 µL of α-amylase was used per 100 g dry corn, as per the manufacturer’s recommendations. The pH was not adjusted in case of amylase corn mix and no external α-amylase was added. The liquefaction was performed at 85 °C for 90 min using Labomat incubator with continuous agitation (Labomat BFA-12, Werner Mathis AG, Switzerland). It is important to note that heating and cooling time (heating and cooling rate of 3 °C/min) were in addition to liquefaction time (90 min).

The pH of the liquefied slurry was adjusted to 4.8 using 10 N sulfuric acid for the SSF process. In control samples, yeast inoculum (2 mL), urea (0.4 mL of 50 % w/v solution), and GA (56.3 µL/100 g dry corn) were added, and the slurry was fermented at 32 °C for 72 h in an automatic incubator (New Brunswick Innova 42R Inc/Ref Shaker, Eppendorf, Connecticut) with continuous agitation at 150 rpm. Yeast inoculum was prepared by mixing 5 g of active dry yeast with 25 mL water and incubated at 32 °C for 20 min. SSF experiments using superior yeast were performed at three GA loadings (0, 25, and 50 % of recommended dosage). The superior yeast was inoculated at the rate of 0.176 g per liter of slurry (~50 µL for 250 mL slurry) as recommended by the manufacturer. Similar to the control experiments, urea solution was used as nitrogen source and slurry was fermented at 32 °C for 72 h in an automatic incubator with continuous agitation at 150 rpm.

To monitor the fermentation, about 2 mL of sample was drawn at 0, 4, 8, 12, 24, 36, 48, and 72 h and centrifuged at 10,000 rpm (Eppendorf Centrifuge 5415 D, Eppendorf AG, Hamburg) for 10 min. The liquid was immediately filtered through 0.2 μm Acrodisc nylon syringe filters (Pall Life Sciences, Port Washington, N.Y.) into HPLC vials. The vials were frozen at −20 °C until further analyzed for sugar and ethanol content.

### Vacuum-assisted fermentation

**Fig. 2:**
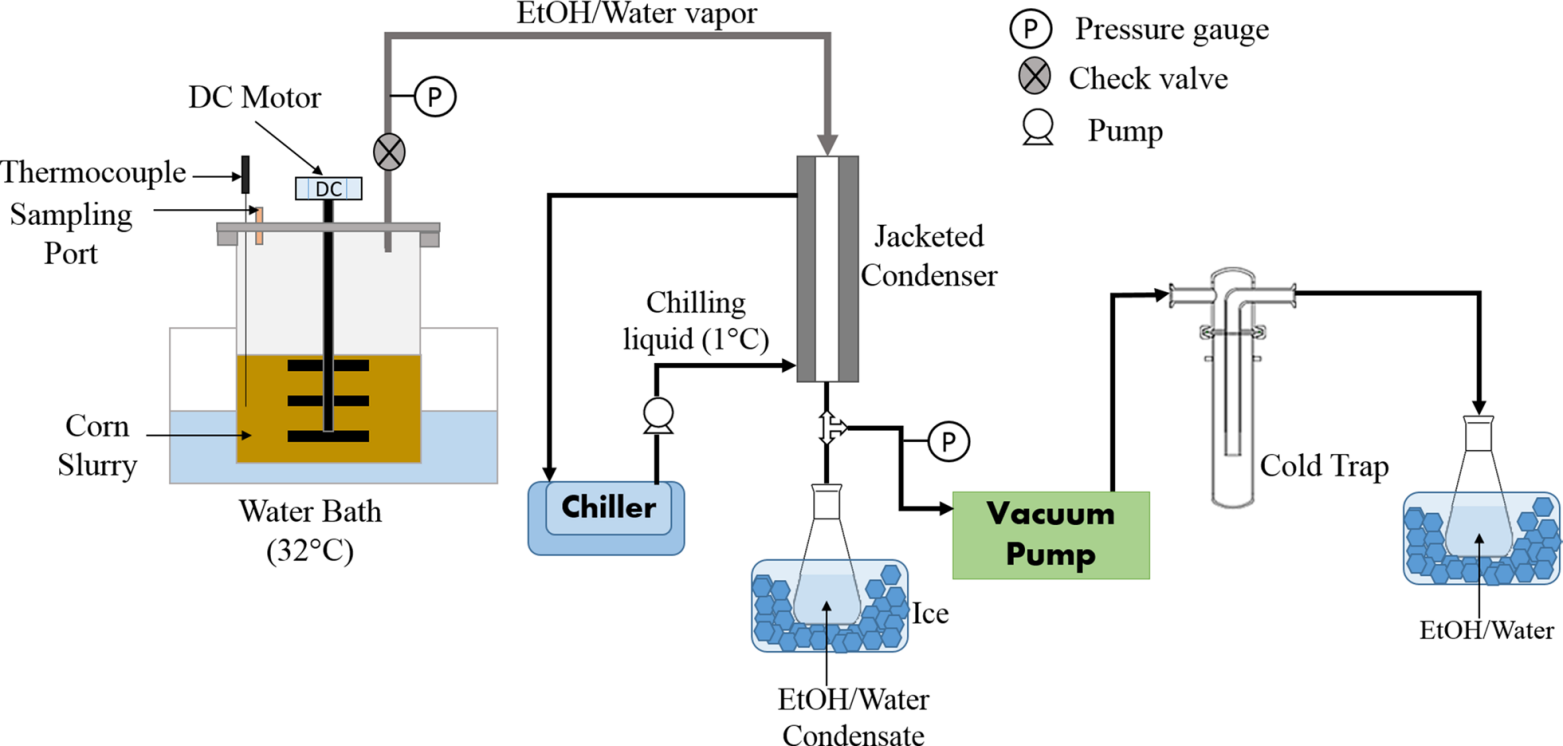
Schematic of lab scale system for the corn fermentation with vacuum stripping system facility. The figure illustrates detail of vacuum-assisted fermentation system used in study

The vacuum-assisted fermentation experiments were performed using a lab scale modified vacuum-reactor system as shown in Fig. 2. It consists of a 3 L modified jacketed fermenter, modified to accommodate thermocouples, agitating motor with stirring blades, and a sampling port. A dry vacuum pump (DryFast model 2044, Welch, Niles, IL) was used to create the vacuum in the fermenter. The system has the facility to condense the evaporated ethanol and water vapors by passing those through a coiled condenser (5977-19, Ace Glass, Vineland, NJ) with chilled liquid circulated at 1 °C. The condensate was collected in a 250 mL conical flask kept under low temperature using ice. For other constructional and operational details of the system, please refer to Huang et al. (2015) [17].

Slurry at 40 % solids was prepared by mixing 500 g (dry basis) of 15 % amylase mix corn with calculated amount of D.I. water. The slurry was liquefied at 85 °C for 90 min in multiple 500 mL stainless steel reactors using Labomat incubator as described in the previous section. The liquefied slurry from multiple reactors was mixed in the 3 L fermenter, and pH was adjusted to 4.8 using 10 N sulfuric acid. The slurry was inoculated with 2 mL urea solution, 0.25 mL superior yeast, and 140.8 µL of glucoamylase (50 % of recommended dose for conventional yeast) and was incubated in water bath set at 32 °C for 72 h. Vacuum pressure at 6.7 kPa (28 in Hg gage) was applied for 1.5 h at 24, 36, 48, and 60 h of the fermentation. The vapors formed due to boiling of slurry were condensed and collected in 250 mL conical flask. A sample was withdrawn from each condensate to determine the ethanol concentration using HPLC. For fermentation profile, about 2 mL of sample was withdrawn at 0, 4, 8, 12, 24, 36, 48, and 72 h of fermentation from the slurry and prepared for HPLC analysis as explained earlier. The samples were also withdrawn after the application of vacuum and analyzed for the sugar and alcohol concentrations.

### Sample analysis (HPLC analysis)

The fermentation samples were analyzed by high-performance liquid chromatography (HPLC; Waters Corporation, Milford, MA) using an ion-exclusion column (Aminex HPX-87H, Bio-Rad, Hercules, CA). The mobile phase was 0.005 M sulfuric acid at 50 °C with a flow rate of 0.6 mL min^−1^. For each sample, a 5 μL injection volume was used with a run time of 30 min. The amounts of sugars, alcohols, and organic acids were quantified using a refractive index detector and using multiple standards.

### Ethanol yields and conversion efficiency

Theoretical ethanol yields were estimated using Eqs. 1 and 2, based on the starch content and free glucose of the corn, assuming complete starch conversion and 100 % fermentation efficiency.

1 $$V_{{{\text{max\_EtOH}}}} = \frac{{W_{\text{C}} *\left( {1 - {\text{MC}}_{\text{C}} } \right)*\left[ {\left( {S*1.11*0.511} \right) + \left( {G*0.511} \right)} \right]}}{{\rho_{\text{EtOH}} }},$$

2 $$E_{{{\text{Th\_EtOH}}}} = \frac{{V_{{{\text{max\_EtOH}}}} }}{{W_{\text{C}} *\left( {1 - {\text{MC}}_{\text{C}} } \right)}},$$

where *V*_max_EtOH_ is the maximum possible volume of ethanol, mL; *W*_C_ is weight of the corn, g; MC_C_ is the moisture content in the corn; *S* is starch content; *G* is free glucose in corn; *ρ*_EtOH_ is density of ethanol, 0.789 g/mL; *E*_Th_EtOH_ is theoretical ethanol yield, L/kg dry corn; 1.11 is the gains during hydrolysis of starch; 0.511 is glucose to ethanol conversion ratio, kg/kg.

Actual ethanol yields were determined by calculating liquid volume in final slurry after 72 h of fermentation. Weight of the final slurry was noted and a sample of the slurry was dried in hot air oven at 105 °C till constant weight achieved (~24 h) to estimate the solid percent in the slurry. The actual ethanol yields were calculated using Eqs. 3, 4, 5.

3 $$W_{\text{L}} = W_{\text{slurry}} *(1 - {\text{Solids}}_{\text{slurry}} ),$$

4 $$V_{\text{EtOH}} = \frac{{W_{\text{L}} }}{{\rho_{{{\text{H}}_{ 2} {\text{O/EtOH}}}} }}*C_{\text{EtOH}},$$

5 $$E_{\text{EtOH}} = \frac{{V_{\text{EtOH}} }}{{W_{\text{C}} *\left( {1 - {\text{MC}}_{\text{C}} } \right)}},$$

where *W*_L_ is the weight of liquid in the fermented slurry, g; *W*_slurry_ is the weight of fermented slurry, g; Solids_slurry_ is the solid fraction in the slurry; *V*_EtOH_ is the volume of ethanol produced, mL; $\rho_{{{\text{H}}_{ 2} {\text{O/EtOH}}}}$ is the density of water–ethanol mixture (g/L) at final ethanol concentration; *C*_EtOH_ is the final ethanol concentration, mL/L; *E*_EtOH_ is the actual ethanol yield, L/kg.

Ethanol conversion efficiencies were calculated by dividing actual ethanol yields with the theoretical ethanol yield (Eq. 6).

6 $$\eta_{\text{EtOH}} = \frac{{E_{\text{EtOH}} }}{{E_{{{\text{Th\_EtOH}}}} }} * 100.$$

### Statistical analysis

The final ethanol concentrations, ethanol yields, starch to ethanol conversion efficiencies, and final glycerol concentrations during various treatments were statistically compared using analysis of variance and Fisher’s least significant difference (SAS version 9.3). The level selected to show the statistical significance in all cases was 5 % (*P* < 0.05).

## Results and discussion

### Comparison of yellow dent corn and amylase mix corn

**Fig. 3:**
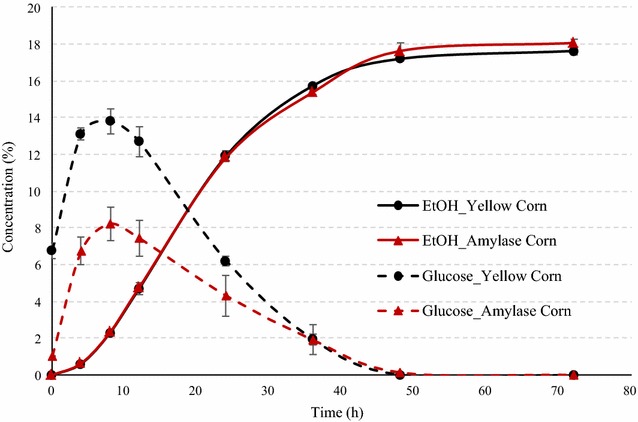
Fermentation profile of dent corn and amylase corn mix. The figure provides the comparison of ethanol concentrations (% v/v) and glucose concentrations (% w/v) during SSF of 100 % dent and 15 % amylase corn mix. The data points in the figure are means of triplicate runs, and error bars represent standard deviations

Ethanol and glucose concentration profiles during fermentation of dent corn and amylase mix corn are illustrated in Fig. 3. After 72 h of fermentation, average final ethanol concentrations for dent corn and amylase mix corn were 17.62 and 18.05 % (v/v), respectively. The small increase in final ethanol concentration for amylase corn could be due to relatively lower glucose inhibition. The peak glucose concentrations for yellow corn were much higher (13.8 %) compared to that from using amylase corn mix (8.22 %). The ethanol yield from amylase corn mix was calculated 0.444 L/kg dry corn (2.98 gal/bu), which was 4.1 % higher than that of dent corn. Most of the fermentation was complete in 48 h for both cases, observed by the small (<0.25 %) amounts of residual glucose, maltose, and maltotriose (Table 1). The results indicated that 15 % addition of amylase corn mixed with conventional corn can eliminate the need of exogenous liquefaction enzyme currently used in the dry-grind process.

**Table 1:** Comparison of sugar concentrations during SSF process among yellow corn and amylase mix corn (mean ± standard deviation of triplicate runs)

Time (h)	Yellow corn				Amylase mix corn			
	Glucose (% w/v)	Maltotriose (% w/v)	Maltose (% w/v)	Fructose (% w/v)	Glucose (% w/v)	Maltotriose (% w/v)	Maltose (% w/v)	Fructose (% w/v)
0	6.76 ± 0.45	1.72 ± 0.23	2.48 ± 0.28	0.15 ± 0.01	1.02 ± 0.02	1.22 ± 0.11	4.73 ± 0.3	0.13 ± 0.01
4	13.1 ± 0.31	1.02 ± 0.25	6.35 ± 0.22	0.44 ± 0.03	6.75 ± 0.74	2.97 ± 0.08	7.23 ± 0.48	0.40 ± 0.02
8	13.8 ± 0.67	0.01 ± 0.01	5.28 ± 0.33	0.41 ± 0.05	8.22 ± 0.94	1.52 ± 0.63	9.63 ± 0.37	0.33 ± 0.02
12	12.69 ± 0.83	0	3.19 ± 0.13	0.35 ± 0.03	7.44 ± 0.95	1.36 ± 2.35	8.96 ± 0.74	0.25 ± 0.02
24	6.21 ± 0.25	0.16 ± 0.02	0.26 ± 0.01	0.2 ± 0.02	4.31 ± 1.1	1.38 ± 0.33	1.65 ± 0.83	0.10 ± 0.02
36	1.93 ± 0.27	0.08 ± 0.002	0.20 ± 0.01	0.14 ± 0.01	1.9 ± 0.81	1.47 ± 0.26	0.19 ± 0.01	0.38 ± 0.52
48	0.01 ± 0.01	0.03 ± 0.003	0.15 ± 0.02	0.08 ± 0.002	0.14 ± 0.03	0.22 ± 0.06	0.18 ± 0.02	0.07 ± 0.01
72	0	0 ± 0.006	0.10 ± 0.02	0.08 ± 0.004	0	0.04 ± 0.002	0.14 ± 0.004	0.08 ± 0.01

### Performance of superior yeast

#### SSF of conventional corn with superior yeast

**Fig. 4:**
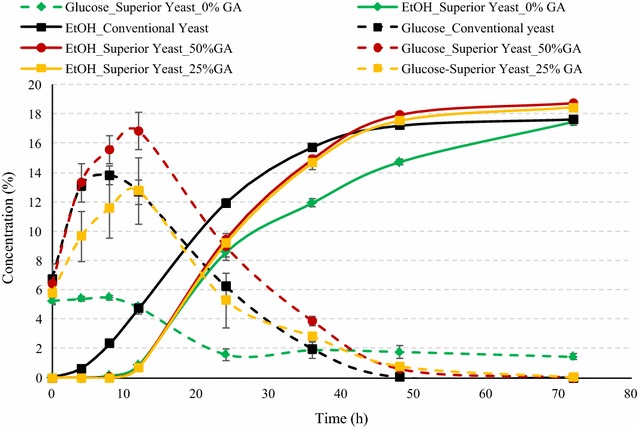
Concentrations of ethanol and glucose during fermentation of yellow dent corn in the conventional dry-grind process by conventional and superior yeast. Figure illustrates the fermentation profile of dent corn mix during SSF by superior yeast at various GA loadings and conventional yeast. *Solid lines* refer to ethanol concentrations (% v/v), and *dotted lines* refer to glucose concentrations (% w/v). The data points in the figure are means of triplicate runs and *error bars* represent standard deviations

**Fig. 5:**
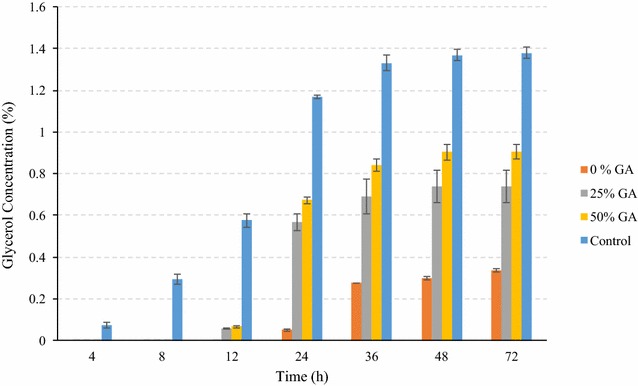
Comparison of glycerol concentration (% w/v) during SSF of dent corn among conventional yeast (control) and superior yeast at various GA loadings. The *bars* in the figure are means of triplicate runs and *error bars* represent standard deviations

The ethanol and sugar production profiles during fermentation of conventional corn using conventional yeast at 100 % GA loading and superior yeast with various glucoamylase loadings are illustrated in Fig. 4. Use of superior yeast even without any addition of glucoamylase (0 %) resulted in similar final ethanol yield as that of control (*P* > 0.05), indicating that superior yeast has sufficient GA expression required to achieve similar ethanol profiles as with control (Table 2). One important factor for these results could be lower substrate inhibition to yeast. The glucose concentrations were relatively low throughout (1.41–5.24 % w/v) the fermentation process for 0 % GA loading, indicating relatively slow conversion of dextrins to glucose, which was simultaneously converted to ethanol by yeast. During initial 12 h of SSF, fermentation rates were very low for superior yeast for all GA loadings. Ethanol concentrations were observed higher by addition of 25 and 50 % GA along with the superior yeast (Fig. 4). Another major reason for high ethanol production using superior yeast was lower levels of glycerol production during fermentation process. The glycerol production was lower in all cases of superior yeast compared to that for conventional yeast (Fig. 5). Glycerol production is considered as an indicator of yeast stress, and typically about 1.2–1.5 % glycerol concentrations are observed in dry-grind ethanol fermentations [19, 20]. In this study, for the superior yeast, maximum glycerol was observed 0.91 % at 50 % GA loading, which was still about 35 % less than that of control. In case of superior yeast use without any addition of GA, final glycerol was observed only 0.34 %, which was about 75 % less than that of control. The ethanol yields of dent corn fermented using superior yeast were in the range of 0.423–0.461 L/kg of dry corn (2.84–3.1 gal/bu). Maximum starch to ethanol conversion efficiency of 88.5 % was observed in case of 50 % GA addition (Table 2). Peak glucose concentration was maximum for superior yeast with 50 % GA addition. In case of superior yeast, it was observed that the peak glucose was observed at 12 h instead of at 8 h as in case of control, indicating relatively slow saccharification initially during SSF.

**Table 2:** Ethanol yields and conversion efficiencies (mean ± standard deviation of triplicate runs)

Conditions	Final ethanol concentration (%)	Final glycerol concentration (%)	Ethanol yield (gal/bu, dry basis)	Conversion efficiency (%)
Conventional corn_ conventional yeast	17.62 ± 0.19 e	1.38 ± 0.03 a	2.86 ± 0.06 d	81.97 ± 1.26 c
Conventional corn_SY_0 % GA	17.46 ± 0.22 e	0.38 ± 0.01 g	2.84 ± 0.03 d	81.30 ± 0.85 c
Conventional corn_SY_25 % GA	18.45 ± 0.22 b c	0.74 ± 0.08 d	3.04 ± 0.03 a b	87.07 ± 0.91 a
Conventional corn_SY_50 % GA	18.73 ± 0.15 a b	0.91 ± 0.04 c	3.09 ± 0.02 a	88.50 ± 0.55 a
15 % Amylase corn_ conventional yeast	18.05 ± 0.23 d	1.24 ± 0.03 b	2.98 ± 0.06 b c	85.07 ± 1.80 b
15 % Amylase corn_SY_0 % GA	16.73 ± 0.06 f	0.30 ± 0.01 g	2.72 ± 0.01 e	77.57 ± 0.33 d
15 % Amylase corn_SY_25 % GA	18.31 ± 0.18 c d	0.54 ± 0.08 f	2.96 ± 0.01 c	84.50 ± 0.33 b
15 % Amylase corn_SY_50 % GA	18.97 ± 0.35 a	0.64 ± 0.06 e	3.05 ± 0.04 a	87.01 ± 1.26 a

*SY* superior yeast

Means followed by the same letter in one column are statistically not different (at *P* < 0.05)

#### SSF of amylase mix corn with superior yeast

**Fig. 6:**
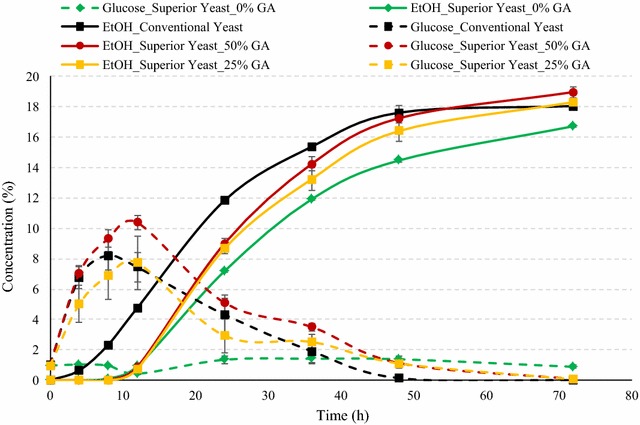
Ethanol and Glucose concentration during SSF of amylase corn mix by conventional and superior yeast. Figure illustrates the fermentation profile of amylase corn mix during SSF by superior yeast at various GA loadings and conventional yeast. The data points in the figure are means of triplicate runs and error bars represent standard deviations. *Solid lines* refer to ethanol concentrations (% v/v), and *dotted lines* refer to glucose concentrations (% w/v)

**Fig. 7:**
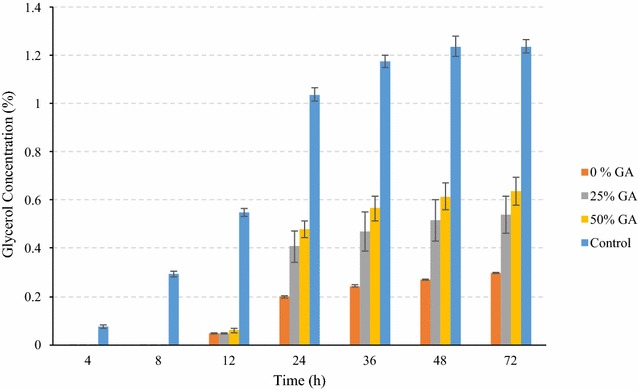
Comparison of glycerol concentration (% w/v) during SSF of amylase corn mix among conventional yeast (control) and superior yeast at various GA loadings. The *bars* in the figure are means of triplicate runs, and *error bars* represent standard deviations

The performance of superior yeast with amylase corn mix was similar to that of conventional corn. The peak glucose during amylase corn mix fermentation using superior yeast was observed at 12 h instead of at 8 h as in case of control, indicating relatively slow conversion (Fig. 6). Compared to those for conventional corn, overall glucose concentrations were low for all GA loadings for amylase corn mix, as observed with the conventional yeast also. Amylase corn mix fermented using superior yeast was considered as control for these experiments. The final ethanol concentration using superior yeast without any addition of GA was about 7.3 % lower than that of control. Addition of only 25 % GA resulted in high ethanol concentration (18.31 %), similar to that of control (18.05 %, using conventional yeast). These results indicate that combined use of amylase corn and superior yeast in the dry-grind process reduced the total external enzyme (α-amylase and glucoamylase) addition by more than 80 %, which would significantly reduce the processing cost. Ethanol concentration as high as 18.7 % was observed at 50 % GA addition along with superior yeast use. At this GA loading, ethanol yield was estimated 0.454 L/kg dry corn (3.05 gal/bu), about 2.35 % higher than that of control. Ethanol conversion efficiencies for amylase mix corn using superior yeast ranged from about 77.57 to 87.01 %. Similar to the case of dent corn, lower levels of glycerol production could have resulted in higher ethanol yields when using superior yeast (Fig. 7). In case of 25 % GA addition with use of superior yeast, final glycerol concentration (0.54 %) was 56.4 % lower than that for conventional yeast (1.24 %). Maximum glycerol concentration of 0.64 % was observed at 50 % GA loading, and was about 49 % less than that of control. The glycerol concentrations in all cases were lower than that of conventional corn.

### Effect of solid loadings

**Fig. 8:**
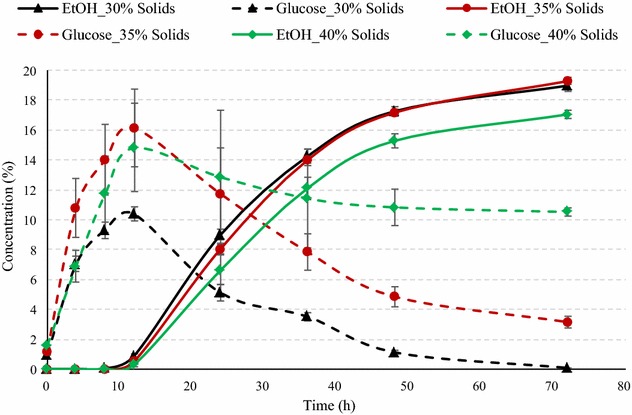
Fermentation profile of amylase corn mix during SSF at various solid loadings. This figure illustrates the effect of solid loadings on the ethanol concentration (% v/v) and glucose concentrations (% w/v) of amylase corn mix during SSF using superior yeast and 50 % GA. The data points in the figure are means of triplicate runs and *error bars* represent standard deviations

To examine the performance of superior yeast at high-solid loadings, amylase mix corn was also liquefied at 35 and 40 % solids, and the slurry was fermented using superior yeast with 50 % GA addition. Figure 8 illustrates the glucose and ethanol concentrations during fermentation at these solid loadings compared to those at 30 % solids. Although final ethanol concentrations at 35 % solids (19.28 %) were higher than that at 30 % solids (18.97 %), however, about 3.14 % glucose remained unconverted after 72 h of fermentation compared to complete conversion at 30 % solids. Final ethanol concentrations at 40 % solids were lower (17.1 %) than both 30 and 35 % solids and 10.5 % of glucose remained unconverted. The ethanol yields for 35 and 40 % solids were 0.358 and 0.268 L/kg dry corn (2.40 and 1.76 gal/bu), respectively, which were 21.14 and 42.0 % lower than that at 30 % solids. High viscosities and yeast stress due to high glucose and ethanol concentration reduce the yeast productivity and result in lower ethanol yields. In this study also, the peak glucose concentrations for 35 and 40 % solids were 1.55 and 1.42 times higher than that at 30 % solids.

### In situ ethanol removal during high-solid SSF

**Fig. 9:**
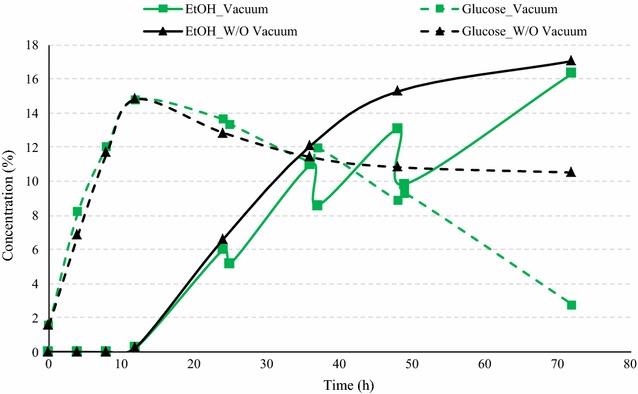
Fermentation profile of amylase corn mix using superior yeast during conventional and vacuum-assisted fermentation (vacuum for 1 h at 24, 36, and 48 h). Figure illustrates the comparison of glucose concentrations (% w/v) and ethanol concentrations (% v/v) during fermentation of amylase corn mix using superior yeast and 50 % GA among conventional and vacuum-assisted fermentation

Simultaneous stripping of ethanol during SSF process can reduce the ethanol inhibition and improve yeast activity. Preliminary experiments were performed to identify the suitable vacuum conditions (vacuum cycles and their frequency) for fermentation at 40 % solids. Application of vacuum for 1 h at 24, 36, and 48 h during fermentation resulted in relatively very high ethanol yields; however, still there were about 2.78 % glucose left unconsumed at the end of fermentation (Fig. 9). Even after removal of significant amount of ethanol during the fermentation process, the final ethanol concentrations were close to that of conventional fermentation (16.33 vs. 17.05 % v/v). Ethanol yield was calculated 0.38 L/kg (2.55 gal/bu), about 44 % higher than that of conventional fermentation at 40 %.

**Fig. 10:**
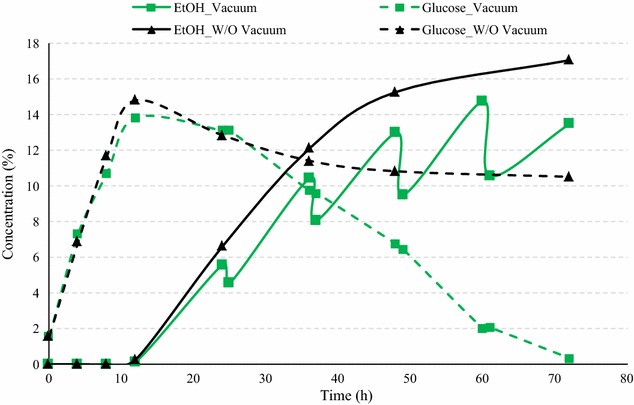
Fermentation profile of amylase corn mix using superior yeast during conventional and vacuum-assisted fermentation (vacuum for 1.5 h at 24, 36, 48, and 60 h). The figure illustrates the comparison of glucose concentrations (% w/v) and ethanol concentrations (% v/v) during fermentation of amylase corn mix using superior yeast and 50 % GA among conventional and vacuum-assisted fermentation

To further improve the fermentation efficiency, another vacuum cycle was added at 60 h and the vacuum time was increased to 90 min. Application of vacuum for 1.5 h at 24, 36, 48, and 60 h during SSF process resulted in complete fermentation compared to 10.5 % residual sugars in case of conventional process (Fig. 10). After vacuum application for 90 min, the ethanol concentrations dropped in the range of 10.4–41.9 mL/L, depending upon the ethanol concentrations at the start of vacuum application. The ethanol drop was higher than those in previous case with 60 min vacuum application (8.2–32.3 mL/L). The final ethanol yield with 82.89 % to ethanol conversion efficiency was estimated 0.433 L per kg dry corn, which was about 1.65 times that for the conventional fermentation at 40 % solids and only 4.6 % lower than that at 30 % solids. Similar results were observed by Shihadesh et al. for dent corn ethanol production using granular starch hydrolyzing enzymes (GSHE) and conventional dry active yeast [13]. The ethanol yields at 40 % solid fermentation with vacuum application produced similar ethanol yields as those of 30 % solids during conventional fermentation.

The ethanol concentrations in the collected condensates ranged from 42.23–71.75 % (v/v), with an average of 57.1 % (v/v). This concentrated ethanol solution can potentially be directly guided to the rectification column during the distillation process for ethanol recovery, which can significantly reduce the energy load on the beer column (first stage of the ethanol recovery process) and overall cost of the dry-grind process.

## Conclusions

Conventional dent corn and amylase mix corn were processed in dry-grind process using superior yeast that expresses glucoamylase and reduces the external enzyme addition. Only 15 % mix of amylase corn was sufficient to eliminate the need of α-amylase addition during liquefaction and achieve similar fermentation profiles. For yellow dent corn, no significant differences were observed in the ethanol yields between the control and using superior yeast without any external addition of glucoamylases. Use of superior yeast can significantly reduce the glucoamylase requirement, improve ethanol yields, and reduce the glycerol production. The vacuum flashing process successfully removed ethanol from the fermentation broth and resulted in complete sugar consumption for 40 % solid slurry. The ethanol yield of 2.9 gal/bu of dry corn with more than 80 % ethanol conversion efficiency was about 65 % higher than that at 40 % solids for conventional fermentation. The study provided a valuable insight about using amylase corn and superior yeast in the dry-grind processing industry and application of vacuum-assisted fermentation to improve fermentation at high solids.

## Abbreviations

*C*_EtOH_: final ethanol concentration
DDGS: distillers dried grains with soluble
D.I.: deionized
*E*_EtOH_: actual ethanol yield
*E*_Th_EtOH_: theoretical ethanol yield
G: free glucose in corn
GA: glucoamylase
GSHE: granular starch hydrolyzing enzymes
MC_C_: moisture content of the corn
S: starch content
SSF: simultaneous saccharification and fermentation
SY: superior yeast
Solids_slurry_: solid fraction in the slurry
*V*_max_EtOH_: maximum possible volume of ethanol
*V*_EtOH_: volume of ethanol produced
*W*_C_: weight of the corn
*W*_slurry_: weight of fermented slurry
*ρ*_EtOH_: density of ethanol
$\rho_{{{\text{H}}_{ 2} {\text{O/EtOH}}}}$: density of water–ethanol mixture
